# Inhibition of mast cell activation by Jaranol-targeted Pirin ameliorates allergic responses in mouse allergic rhinitis

**DOI:** 10.1515/biol-2022-1030

**Published:** 2025-03-28

**Authors:** Yue Huang, Shuhua Su, Bo Duan, Yunfei Zhang, Zhengmin Xu

**Affiliations:** Department of Otolaryngology-Head and Neck Surgery, Children’s Hospital of Fudan University, No 399 Wanyuan Road, Shanghai, China; Department of Otolaryngology, Qinzhou Maternity and Child Health Care Hospital, Qinzhou, Guangxi Province, China

**Keywords:** Jaranol, Kumatakenin, allergic rhinitis, Pirin

## Abstract

Jaranol, a bioactive compound derived from various traditional medicinal herbs, has demonstrated significant anti-inflammatory properties. This study investigates its effects and possible mechanisms underlying its anti-inflammatory role in mast cells, as well as ovalbumin (OVA)-induced allergic rhinitis (AR) mice model. Forty mice were randomly divided into blank, AR, dexamethasone (positive control), and Jaranol groups (10 mg/ml), with 10 mice in each group. Jaranol was found to inhibit nasal mucosal inflammation and reduce mast cell numbers in AR models. It also inhibited the secretion of several inflammatory cytokines (IFN-γ, TNF-α, IL-1β, IL-6, MCP-1, and CXCR10) from mast cells, as well as mast cell proliferation and migration. Interestingly, Pirin was differentially expressed in blank, AR, and Jaranol groups. Further studies indicated that Jaranol inhibited inflammatory cytokine secretion from mast cells by mediating Pirin and also inhibited mast cell proliferation and migration. Moreover, it inhibited mast cell function by suppressing Pirin expression. These findings suggest that Jaranol exerts its therapeutic effects by inhibiting Pirin expression in mast cells, thereby reducing inflammation and histopathological changes associated with AR.

## Graphical Abstract

**Figure:**
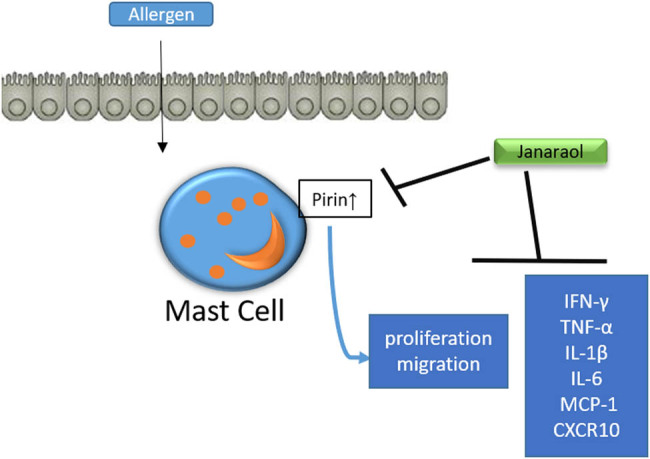

## 1 Introduction

Allergic rhinitis (AR) is a chronic inflammation of the nasal mucosa that significantly impairs the quality of life for millions worldwide [1,2]. It is triggered by an IgE-mediated hypersensitivity reaction to environmental allergens, which causes symptoms such as sneezing, stuffy nose, itching, and runny nose [3,4]. The pathogenesis of AR involves a complex interplay of immune cells, among which mast cells play a pivotal role [5,6]. When exposed to allergen, mast cells release a variety of inflammatory mediators, including histamines [7,8], cytokines [9], and proteases [10,11], leading to AR symptoms and histopathological changes in the nasal tissue.

The nasal administration of Chinese herbal medicine involves applying herbal preparations directly into the nasal cavity, where they are absorbed through the nasal mucosa to provide local or systemic therapeutic effects. This method includes dosage forms such as nasal drops, rinses, emulsions, aerosols, sprays, powders, inhalants, gels, and films. Compared to traditional forms like injections or oral medications, nasal administration of Chinese herbal medicine offers advantages like ease of use, targeted delivery, high safety, and fast absorption. Nasal drop is the commonly used nasal formulation; it can treat various diseases, such as nasal disorders, cardiovascular diseases, cerebrovascular diseases, colds, and migraines. Liu et al. [12] found that Yu-ping-feng nasal drops could treat AR in the rat model. Yip et al. [13] used network pharmacology analysis to test common gene targets in coronavirus disease 2019 (COVID-19) and confirmed that allergic rhinitis nasal drops have an inhibitory effect on viral infection; moreover, it also reduced the inflammatory response post-infection and potentially lowered the risk of lung fibrosis. Fan et al. [14] conducted a dose-escalation study comparing lentinan nasal drops to a placebo to assess the safety and efficacy of lentinan nasal drops in COVID-19 patients; results indicated that lentinan nasal drops are safe and well-tolerated and can shorten viral clearance time. Qian et al. [15] explored the effects of ketamine nasal drops on postoperative pain in children after cold plasma ablation tonsillectomy; results indicated that ketamine nasal drops are safe, reduce pain, and shorten recovery time. In sum, nasal drops have distinct advantages for treating nasal and pulmonary diseases over traditional formulations (such as granules, pills, and injections), making nasal drop development highly promising.

Pirin, a nuclear protein, has been identified as a significant regulator in the inflammatory response [16] and mast cell activation [17]. Elevated levels of Pirin are associated with increased inflammatory activity, suggesting that targeting Pirin expression may provide a novel therapeutic approach for managing AR. Despite its potential, the modulation of Pirin in allergic conditions remains underexplored. Jaranol, also called Kumatakenin, could be extracted from various traditional medicinal herbs, such as licorice, *Psychotria serpens*, and *Siparuna cristata* [18,19]. Previous studies have demonstrated the efficacy of Jaranol in inhibiting SARS-CoV-2 replication [20], exerting anti-cancer [21], alpha-glucosidase inhibitory [22], anti-bacterial [23], anti-oxidant [24], and anti-inflammatory activities [25]. However, the specific effects of Jaranol on Pirin expression in mast cells and its consequent impact on AR have not been thoroughly investigated.

The main objective of this study is to thoroughly investigate the mechanism of action of Jaranol in the treatment of AR, to provide an experimental basis for the discovery of target proteins and pathways regulating AR, and to provide a treatment for AR.

## 2 Materials and methods

### 2.1 Reagents and experimental animals

Jaranol was obtained from Chengdu Push Biotechnology Co., Ltd (China). Ovalbumin (OVA) was provided by MCE (MedChemExpress, USA), and aluminum hydroxide adjuvant and dexamethasone were provided by Suzhou Boao Long Technology Co., Ltd. (China), recombinant anti-mast cell tryptase antibody, anti-Ki67 primary antibody, 4',6-diamidino-2-phenylindole (DAPI) staining solution, crystal violet staining solution, cell counting kit-8 (CCK-8) assay kit, reverse transcription and real-time polymerase chain reaction (PCR) assay kit, and western blot-related reagents were obtained from Wuhan Servicebio Technology Co., Ltd. (China). The enzyme-linked immunosorbent assay (ELISA) kit was purchased from Abcam (USA). Pirin interference virus and overexpression virus were obtained from Shanghai Jima Gene (China). P815 mast cells were provided by the Cell Bank of the Chinese Academy of Sciences. The specific experimental procedures involving the above reagents were performed according to the instructions provided with each assay kit.

A total of 40 BALB/c mice, with equal numbers of males and females, were purchased for this study. All animals were SPF level (Shanghai SLAC Laboratory Animal Co., Ltd., China), aged 6–8 weeks, and weighed 20 ± 2 g. All animals were kept in a controlled environment with 50% humidity and 20°C temperature, with a 12-h light/dark cycle. The water and standard feed were sterilized by high pressure, and the mice had free access to them.


**Ethical approval:** The research related to animal use has been complied with all the relevant national regulations and institutional policies for the care and use of animals and has been approved by the Animal Ethics Committee of the Children’s Hospital affiliated with Fudan University.

### 2.2 Modeling methods and experimental groups

Sensitization phase: Mice were raised in SPF-level animal facilities and allowed to acclimate for one week before the experiment. A 1% solution of OVA was prepared by dissolving 10 mg of OVA in 1 ml of saline and then mixed with an equal volume of a 2% aluminum hydroxide adjuvant. The mixture was shaken for 30 min. Each mouse received an intraperitoneal injection of 200 µl of this solution daily for 14 consecutive days. Challenge phase: After 14 days of systemic sensitization, intranasal instillation was initiated using a 5% OVA solution (50 mg OVA in 1 ml of saline). Each nasal cavity received 50 µl of the solution once a day for 14 days. On the final day, mice were observed within 10 min of nasal instillation for symptoms of nasal itching, sneezing, and runny nose. The symptoms of AR in mice were scored according to Table 1, with a score exceeding 5 indicating a successful model.

**Table 1:** Scoring criteria for symptoms of AR in mice

Symptom	Score 1	Score 2	Score 3
Nasal itching	Scratching nose 1–2 times	Between the two	Continuous scratching of the nose
Sneezing	1–3 times	4–10 times	>11 times
Runny nose	Flowing to the anterior nares	Extending beyond the anterior nares	Covering the whole face

Forty mice were randomly divided into four groups: blank group, model group, dexamethasone group (positive control), and Jaranol group, with 10 mice in each group, with an equal number of males and females. (1) Blank group: 50 µl of saline was instilled into the nasal cavity during the sensitization stage, and no drug treatment was administered. (2) model group: 50 µl of 5% OVA was instilled into the nasal cavity during the sensitization stage, and no drug treatment was administered. (3) Dexamethasone group: 50 µl of 5% OVA was instilled into the nasal cavity during the sensitization stage, and dexamethasone saline solution (200 µl, 500 µg/ml) was injected intraperitoneally at the same time. (4) Jaranol group: 50 µl of 5% OVA was instilled into the nasal cavity during the sensitization stage, and 50 µl of 10 mg/ml Jaranol was also instilled in the nasal cavity at the same time. This study was approved by the Ethics Committee of Xinhua Hospital and attached to Shanghai Jiao Tong University School of Medicine (XHEC-NSFC-2020-097). The research related to animal use has been complied with all the relevant national regulations and institutional policies for the care and use of animals.

### 2.3 Hematoxylin and eosin (HE) staining, immunohistochemical staining, and immunofluorescence staining

After 12 h following the last administration, mice were anesthetized with 2% pentobarbital sodium solution and euthanized. The mouse heads were decalcified with 10% ethylenediaminetetraacetic acid, and then, tissue samples were collected from three sites (the posterior part of the upper incisor, the incisor papilla, and the second palatal ridge). Tissues were soaked in 80% ethanol for 24 h, then dehydrated, cleared, and embedded in paraffin using a dehydration machine. The thickness of the sections was 5 μm.

(1) HE staining: The paraffin sections were first dewaxed in xylene for 5 min, repeated three times. The sections were then sequentially immersed in anhydrous ethanol, 95% ethanol, and 75% ethanol for 5 min each. After rinsing with distilled water for 10 min, the sections were stained with hematoxylin for 5 min, with staining intensity observed under a microscope and ethanol differentiation time adjusted as needed. The sections were then stained with 0.5% eosin solution for 10–15 s, rinsed with distilled water, and observed under a microscope. Finally, the sections were dehydrated in 75, 95, and 100% ethanol, cleared in xylene, and sealed with neutral gum.
(2) Immunohistochemical staining: The dewaxing and hydration steps were the same as for HE staining. For antigen retrieval, sections were heated at 95°C in antigen retrieval solution (Servicebio) for 15 min and then cooled to room temperature for 30 min. Sections were incubated with immunostaining blocking solution (Servicebio) at room temperature for 1 h. After removing the blocking solution, primary antibodies (anti-Ki67 primary antibody, 1:1,000, anti-Mast Cell Chymase Rabbit pAb, 1:1,000; Servicebio) were added and incubated overnight at 4°C. The sections were washed with phosphate buffer saline (PBS) three times for 10 min each, followed by the addition of the appropriate secondary antibodies and incubation at 37°C for 1 h. After washing with PBS again, 3,3'-diaminobenzidine staining (Servicebio) was performed. Positive staining appeared brown under a microscope. Nuclear staining with hematoxylin followed along with gradient alcohol dehydration, xylene transparency, and neutral gum sealing.
(3) Immunofluorescence staining: The steps of dewaxing, gradient ethanol hydration, and antigen retrieval were the same as for immunohistochemical staining. After blocking, primary antibodies (Servicebio) were added and incubated overnight at 4°C. The sections were washed with PBS three times for 10 min each, followed by the addition of immunofluorescence secondary antibodies and incubation at room temperature for 2 h. After washing with PBS again, the sections were sealed with a mounting medium containing DAPI, and the fluorescence staining was observed under a fluorescence microscope.

### 2.4 Cell culture and virus infection

P815 mast cells were obtained from the Cell Bank of the Chinese Academy of Sciences. The P815 cells were cultured in McCoy’s 5A medium (Servicebio) supplemented with 10% fetal bovine serum (Sigma). The medium was replaced every other day, and the cells were passaged and seeded into appropriate culture vessels according to their growth conditions. For Pirin interference and overexpression experiments, P815 cells were seeded in 6 cm culture dishes (Corning) and infected with the virus once cell density reached approximately 70%. Six micrograms per milliliter of polybrene and 1 × 10^6^ PFU of virus were added to each well, and the cells were incubated at 37°C for 3 days. Infection efficiency was observed using a fluorescence microscope, and interference and overexpression were confirmed by western blot. Compound 48/80 (10 μg/ml) (MedChemExpress) was used to activate P815 cells *in vitro* as a model group, and the concentration of Jaranol for *in vitro* treatment was 10 μg/ml.

### 2.5 Real-time PCR

RNA was first extracted from P815 cells seeded in 6 cm culture dishes. One milliliter of TRIzol was added to each dish, and the cells were collected and transferred into 1.5 ml eppendorf (EP) tubes. Two hundred microliters of chloroform was added, mixed, and left at 4°C for 10 min. The mixture was centrifuged at 12,000 rpm for 15 min at 4°C, and the upper aqueous phase was transferred to a new, RNase-free EP tube. Five hundred microliters of pre-cooled isopropanol was added, and the mixture was inverted and mixed. After standing at 4°C for 10 min, the mixture was centrifuged at 12,000 rpm for 10 min. The supernatant was discarded, and the RNA precipitate at the bottom of the tube was visible. After washing with 75% ethanol and air drying at room temperature, the RNA turned from white to a colorless, transparent state. Finally, 30 µl of diethyl pyrocarbonate water was added to dissolve the RNA, which was stored at −80°C. Quantitative PCR detection was performed using the 2 × Fast SYBR Green qPCR Master Mix kit (Servicebio) with a 20 μl reaction system. Following an initial denaturation at 95°C for 30 s, amplification was performed for 45 cycles at 95°C for 5 s and 60°C for 45 s. The relative gene expression levels were calculated according to the formula 2^−ΔΔCT^ and compared between groups.

The primer sequences were as follows:

IFN-r forward: 5′-TGAATGTCCAACGCAAAGCA-3′;

    reverse: 5′-TCGACCTCGAAACAGCATCT-3′;

TNFa forward: 5′-TTGGAACTTGGAGGGCTAGG-3′;

    reverse: 5′-CACTAAGGCCTGTGCTGTTC-3′;

IL-1β forward: 5′-GAAATGCCACCTTTTGACAGTG-3′;

    reverse: 5′-TGGATGCTCTCATCAGGACAG-3′;

IL-6 forward: 5′-CTGCAAGAGACTTCCATCCAG-3′;

    reverse: 5′-AGTGGTATAGACAGGTCTGTTGG-3′;

MCP-1 forward: 5′-TAAAAACCTGGATCGGAACCAAA-3′;

    reverse: 5′-GCATTAGCTTCAGATTTACGGGT-3′;

CXCR10 forward: 5′-CCAAGTGCTGCCGTCATTTTC-3′;

    reverse: 5′-TCCCTATGGCCCTCATTCTCA-3′;

GAPDH forward: 5′-AGGTCGGTGTGAACGGATTTG-3′;

    reverse: 5′-TGTAGACCATGTAGTTGAGGTCA-3′.

### 2.6 ELISA

The supernatant from the P815 cell culture was collected and centrifuged at 1,500 rpm for 10 min to detect the expression levels of IFN-γ, TNF-α, IL-6, and IL-1β. The assay was performed using the Abcam ELISA kit. In brief, standard samples were prepared and added to the ELISA detection plate. The test samples were diluted 2, 5, and 10 times and added to the plate, while sample dilution buffer was added to the blank wells, with 100 μl of diluted sample in each well. The plate was sealed, shaken at 100–300 rpm at room temperature for 2 h, and then washed five times by adding 300 μl of washing solution to each well, leaving it for 5 min. After removing the residual liquid, 100 μl of 1× antibody working solution was added to each well. Following a repeat wash, 100 μl of HRP-conjugated secondary antibody solution was added to each well and incubated for 30 min at room temperature. After washing, 90 μl of TMB substrate solution was added to each well, and the plate was incubated in the dark at room temperature for 10 min. Finally, 50 μl of stop solution was added to each well to terminate the reaction. Absorbance at 450 nm was measured using an enzyme immunoassay instrument, and the results were compared between groups.

### 2.7 CCK-8 assay

The cells were seeded at a density of 5 × 10³ cells per well in a 96-well cell culture plate (Corning Inc.) with 200 μl of culture medium per well. A blank control well (without cells) was also included. After incubating in a cell culture incubator for 24 h, 20 μl of CCK-8 solution (Servicebio) was added to each well, and the plate was incubated for an additional 2 h. The absorbance at 450 nm was measured using an enzyme immunoassay instrument, and OD values were compared between groups.

### 2.8 Transwell assay

The migration of P815 cells was measured using a 24-well Transwell chamber (Corning Inc.) with a 6.5 mm polycarbonate membrane (5 μm pore size). A fresh culture medium containing P815 cells (5 × 10⁴ cells/well) was added to the upper chamber, and the same culture medium was added to the lower chamber. After a 24-h incubation, the fetal bovine serum concentration in the lower chamber was adjusted to 20%, and incubation continued for an additional 24 h. Afterward, non-migratory cells on the membrane surface were removed with a cotton swab. Cells were then fixed with 4% paraformaldehyde at room temperature for 15 min, washed three times with PBS for 10 min each, and stained with crystal violet for 10 min. The number of migrated cells was observed under an optical microscope. Ten random fields were selected under a bright-field microscope to count the cells on the underside of the membrane, and the average cell count was taken for comparison between groups.

### 2.9 Western blot analysis and proteomics

Protein extraction buffer (containing protease inhibitors) was added to the 6 cm culture dish containing P815 cells, which were collected and kept at 4°C for 10 min. The cells were then sonicated on ice at 30 W for 2 min and left at room temperature for 15 min. The mixture was centrifuged at 12,000 rpm for 15 min at 4°C, and the supernatant was transferred to a new centrifuge tube. This step was repeated with a second centrifugation under the same conditions, and the supernatant was collected. The protein concentration was determined using the BCA protein quantification kit (Servicebio), after which 5× loading buffer was added, and the mixture was heated at 100°C for 5 min to denature the proteins. Sodium dodecyl sulfate-polyacrylamide gel electrophoresis was performed using a 10% gel, with 30 μg of protein loaded per lane. After electrophoresis, the proteins were transferred onto a PVDF membrane, which was blocked at room temperature for 30 min. The membrane was incubated overnight at 4°C with the following primary antibodies: Pirin (goat anti-rabbit, 1:1,000, Servicebio), p-p65 (goat anti-rabbit, 1:1,000, CST), p65 (goat anti-rabbit, 1:1,000, CST), IκBα (goat anti-rabbit, 1:1,000, CST), and β-actin (goat anti-mouse, 1:2,000, CST). After three washes with TBS-tween (Servicebio) for 15 min each at room temperature, the membrane was incubated with the corresponding HRP-conjugated secondary antibody at room temperature for 2 h. Following another set of three washes with TBS-Tween for 10 min each, ECL solution was added, and the PVDF membrane was placed in an automated chemiluminescence imaging system for image acquisition and analysis. The proteomics experiment was conducted by Shanghai Aiputekang Biotechnology Co., Ltd. (China), and four tissue samples from each group were used for proteomic analysis.

### 2.10 Statistical analysis

The results were expressed as means ± standard deviation. A Student’s *t*-test was used to compare individual data with control values. *p* < 0.05 was considered statistically significant.

## 3 Results

### 3.1 Jaranol inhibited nasal mucosal inflammation and reduced mast cell numbers in a mouse model of rhinitis

**Figure 1:**
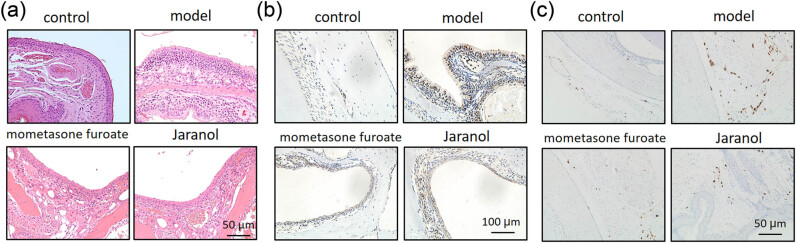
Jaranol inhibits nasal mucosal inflammation in a mouse model of rhinitis. (a) HE staining shows successful preparation of a mouse model of rhinitis induced by OVA, and Jaranol and positive control dexamethasone significantly inhibit nasal mucosal damage and inflammation in the model mice. (b) Ki67 immunohistochemistry staining shows that Jaranol inhibits cell proliferation at the site of rhinitis in the model mice. (c) Recombinant Anti-Mast cell tryptase antibody immunohistochemistry staining shows that Jaranol reduces the number of mast cells at the site of nasal mucosal damage. Scale bar: 50 or 100 μm.

To determine whether Jaranol can alleviate the inflammatory response in rhinitis, we established a rhinitis model in BALB/c mice using OVA nasal drops. As shown in Figure 1, the control group had intact nasal mucosal tissue with columnar epithelium, while the model group (Figure 1, model) had significantly thicker nasal mucosal epithelium with numerous infiltrating inflammatory cells, goblet cell metaplasia, hypertrophy, and increased mucosal interstitial edema. Dexamethasone served as the positive control drug. After treatment with dexamethasone, nasal mucosal hyperplasia and inflammatory cell infiltration were reduced (Figure 1, dexamethasone). Treatment with Jaranol significantly reduced the thickness of the nasal mucosa and decreased inflammatory cell infiltration. The pathological morphology was similar to that of the dexamethasone group (Figure 1, Jaranol). These findings suggest that Jaranol inhibits nasal mucosal damage and inflammation in the mouse model of rhinitis.

Next, we detected the expression of Ki67 (proliferating cell nuclear antigen) in the nasal mucosa of each group. As shown in Figure 1b, the model group showed significantly higher Ki67 expression compared to the control group. After treatment with dexamethasone and Jaranol, Ki67 expression was significantly decreased, indicating that Jaranol inhibits cell proliferation at the site of rhinitis in mice.

Mast cells are the main receptor cells involved in the pathogenesis of AR, triggering early symptoms by releasing substances such as histamine, vascular relaxing peptide, tryptase, and arachidonic acid derivatives. Therefore, we observed the positive staining of mast cells in nasal mucosal tissue. As shown in Figure 1c, mast cell numbers were low in the control group, while significantly increased in the model group. Treatment with mometasone furoate and Jaranol significantly reduced mast cell numbers compared to the model group, indicating that Jaranol can decrease mast cell numbers in the nasal mucosa of mice with AR.

### 3.2 Jaranol inhibited inflammatory cytokine secretion from mast cells

**Figure 2:**
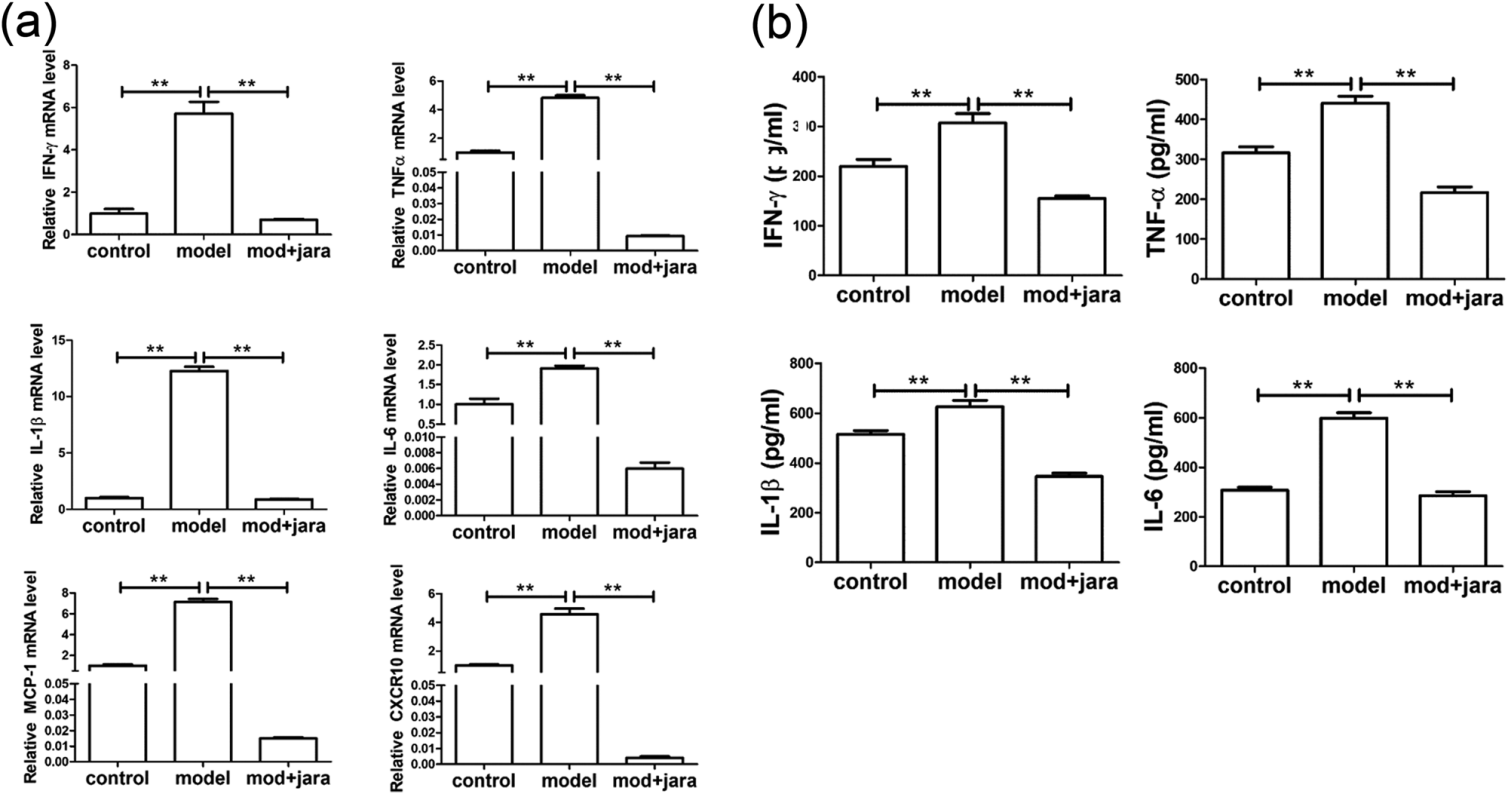
Jaranol inhibits inflammatory cytokine secretion from mast cells. (a) Real-time PCR shows that Jaranol inhibits the mRNA expression of IFN-γ, TNF-α, IL-1β, IL-6, MCP-1, and CXCR10 in mast cells. (b) ELISA shows that Jaranol inhibits the secretion of IFN-γ, TNF-α, IL-1β, and IL-6 from mast cells. Mod: model; jara: Jaranol. **p < 0.01.

Based on the above results, we further investigated whether Jaranol could alleviate rhinitis symptoms in mice by inhibiting mast cell infiltration and the secretion of inflammatory cytokine secretion. Therefore, we used the P815 mouse mast cell line *in vitro* to observe whether Jaranol could reduce inflammatory cytokine secretion from P815 cells. As shown in Figure 2a, real-time PCR results showed that compared to the control group, mRNA levels of IFN-γ, TNF-α, IL-1β, IL-6, MCP-1, and CXCR10 were significantly increased in the model group, while treatment with Jaranol significantly decreased the expression of these inflammatory cytokines. ELISA results further confirmed that Jaranol inhibits the secretion of inflammatory cytokines IFN-γ, TNF-α, and IL-1β from mast cells (Figure 2b).

### 3.3 Jaranol inhibited mast cell proliferation and migration

**Figure 3:**
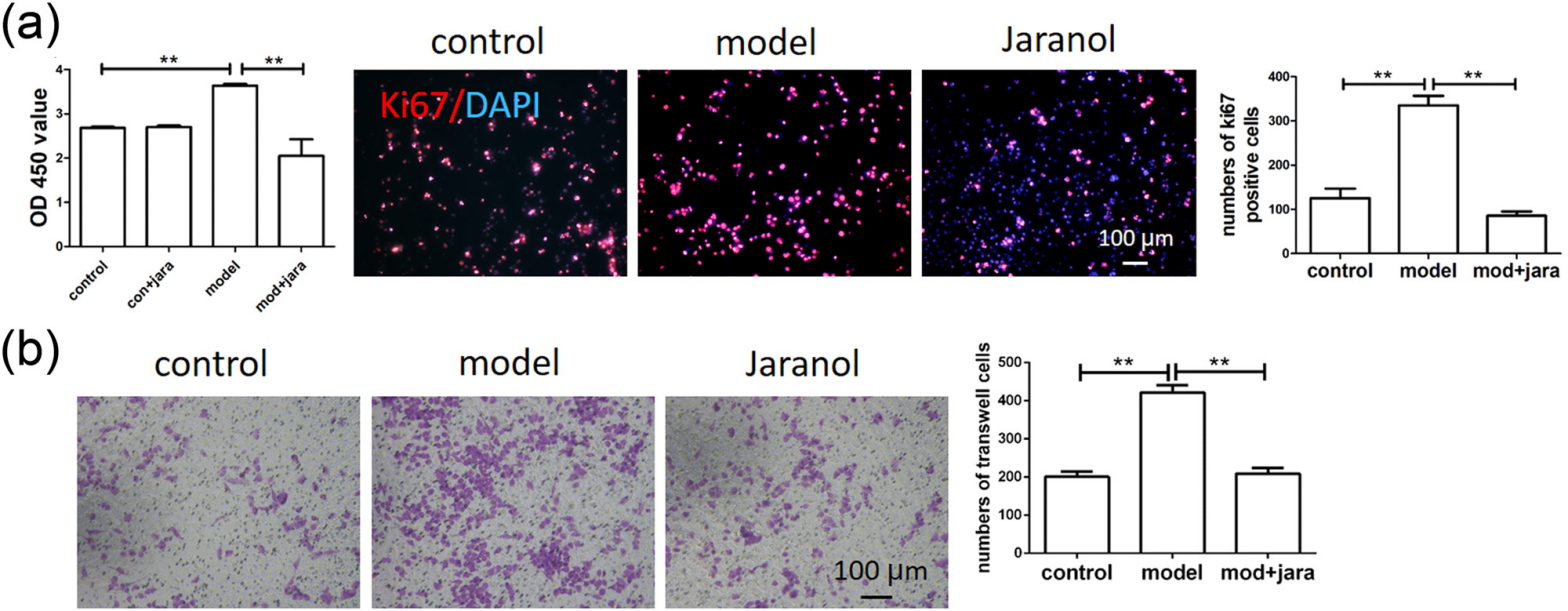
Jaranol inhibits mast cell proliferation and migration. (a) CCK-8 and Ki67 immunofluorescence staining shows that Jaranol inhibits mast cell proliferation; (b) transwell experiment shows that Jaranol inhibits mast cell migration. Mod: model; jara: aranol. Scale bar: 50 or 100 μm. **p < 0.01.

We used the CCK-8 assay to detect mast cell proliferation and found that Jaranol did not affect normal mast cell proliferation but significantly inhibited proliferation in the model group. The results of Ki67 immunofluorescence staining were consistent with those of the CCK-8 assay. In this experiment, the Ki67-positive signals appeared in red, showing significantly increased Ki67 expression in the model group, while Jaranol significantly downregulated Ki67 expression, thus inhibiting mast cell proliferation (Figure 3a). In rhinitis, mast cells in the lamina propria can migrate toward the mucosal surface due to repeated allergen exposure. We observed the effect of Jaranol on mast cell migration using a transwell assay and found that the migration ability of mast cells in the model group was significantly enhanced compared to the control group, while Jaranol inhibited this phenomenon (Figure 3b).

### 3.4 Proteomic sequencing screened differential expression and functional genes in mouse nasal mucosal tissue

**Figure 4:**
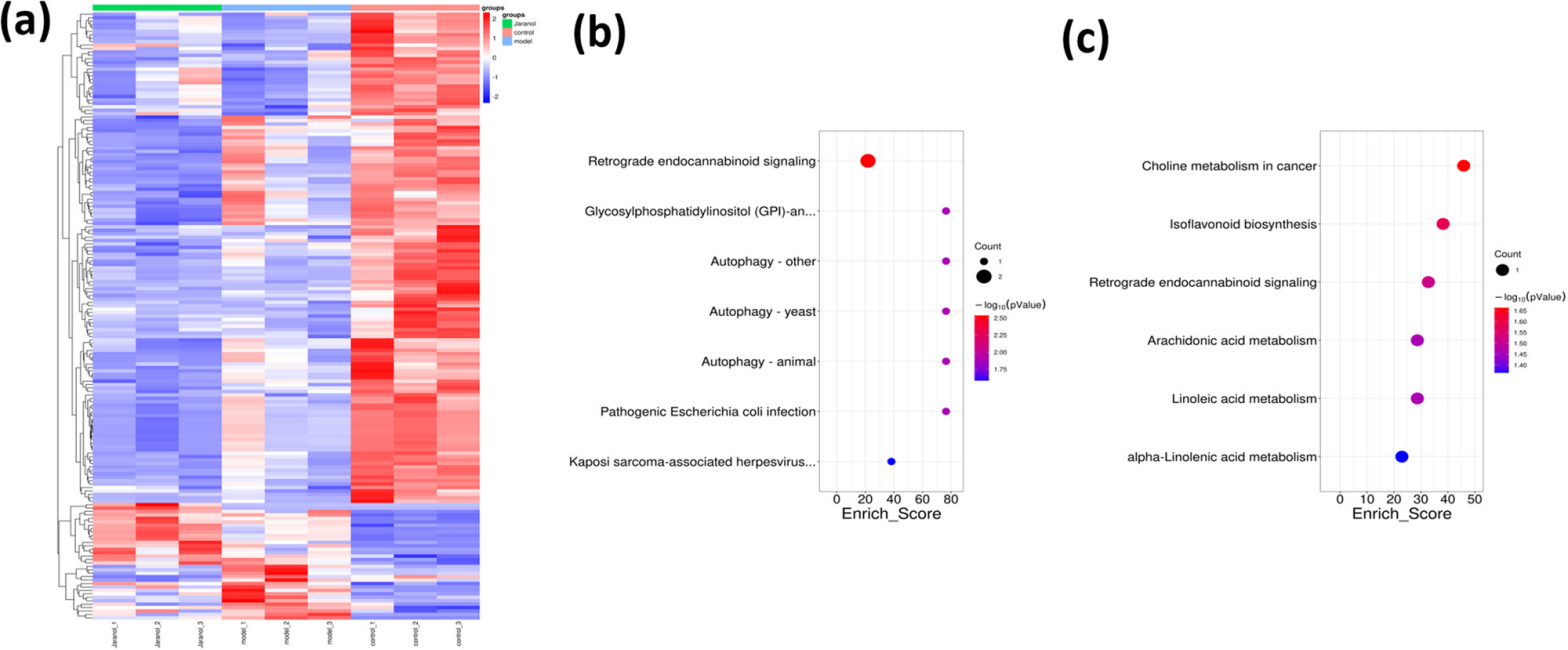
Differential expression and functional genes screened by proteomic sequencing of mouse nasal mucosal tissue in each group. (a) Heatmap of differentially expressed proteins among the three groups; (b) GO analysis chart of differentially expressed proteins; (c) KEGG analysis chart of differentially expressed proteins.

We conducted proteomic sequencing on mouse nasal mucosal tissue and identified 102 differentially expressed proteins in the model group compared to the control group. Additionally, there were 54 differentially expressed proteins between Jaranol and model groups, and 26 common differentially expressed proteins among the three groups, including 15 upregulated and 11 downregulated proteins. From these 26 proteins, we selected Pirin, which may be involved in regulating mast cell function, to further investigate its role in mast cell function and rhinitis pathogenesis. Figure 4a shows a heatmap of the common proteins expressed across the three groups, with red indicating upregulation and blue indicating downregulation. Figure 4b shows a line graph of the expression trends of each group’s proteins. It also shows the gene ontology (GO) analysis chart, indicating the molecular function, biological process, and cellular component of differentially expressed proteins. Figure 4b shows the Kyoto encyclopedia of genes and genomes (KEGG) analysis chart, showing various functions of differentially expressed proteins.

### 3.5 Jaranol inhibited inflammatory cytokine secretion from mast cells by mediating Pirin

**Figure 5:**
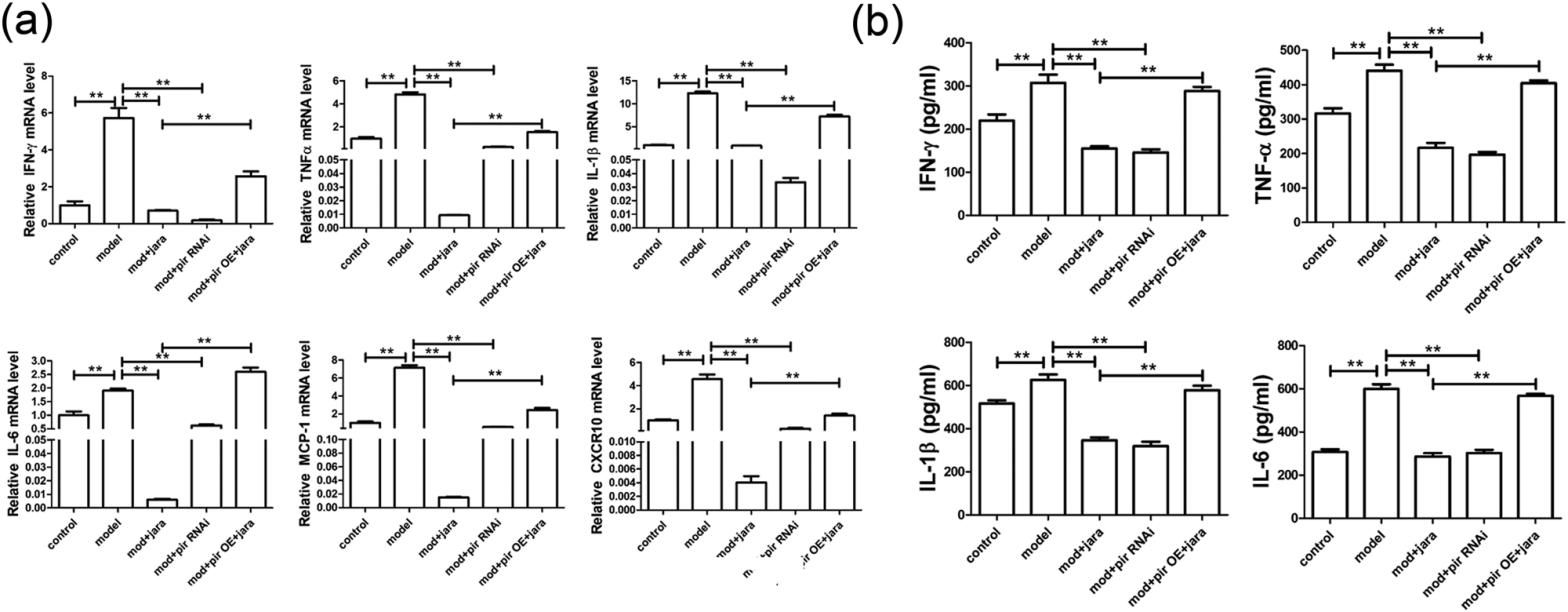
Jaranol inhibits inflammatory cytokine secretion from mast cells by acting on Pirin. (a) Real-time PCR shows that both Jaranol and interference with Pirin can inhibit the mRNA expression of IFN-γ, TNF-α, IL-1β, IL-6, MCP-1, and CXCR10 in mast cells, and the inhibitory effect of Jaranol on cytokine secretion is weakened in mast cells overexpressing Pirin. (b) ELISA shows that both Jaranol and interference with Pirin can inhibit the secretion of IFN-γ, TNF-α, IL-1β, and IL-6 from mast cells, and the inhibitory effect of Jaranol on cytokine secretion is weakened in mast cells overexpressing Pirin. Pir: Pirin; RNAi: interference; OE: overexpression. **p < 0.01.

As shown in Figure 5a, the model group exhibited increased mRNA expression levels of IFN-γ, TNF-α, IL-1β, IL-6, MCP-1, and CXCR10 compared to the control group. However, after Jaranol treatment, these expression levels significantly decreased. When Pirin was silenced in mast cells, mRNA levels of these inflammatory cytokines were also significantly reduced. In contrast, when Jaranol was added to mast cells overexpressing Pirin, cytokine mRNA expression increased compared to the Jaranol-treated group, suggesting that Jaranol inhibited the Pirin-promoted expression of inflammatory cytokines at the mRNA level (Figure 5a). Similarly, Figure 5b shows ELISA results of IFN-γ, TNF-α, IL-1β, and IL-6, indicating that the secretion of these inflammatory cytokines increased in the model group. Both Jaranol treatment and Pirin silencing inhibited the secretion of these cytokines in mast cells. However, in mast cells with overexpressed Pirin treated with Jaranol, Jaranol still inhibited the secretion of inflammatory cytokines promoted by Pirin. These results suggest that Jaranol may regulate mast cell function by regulating the Pirin expression.

### 3.6 Jaranol inhibited mast cell proliferation and migration through Pirin

**Figure 6:**
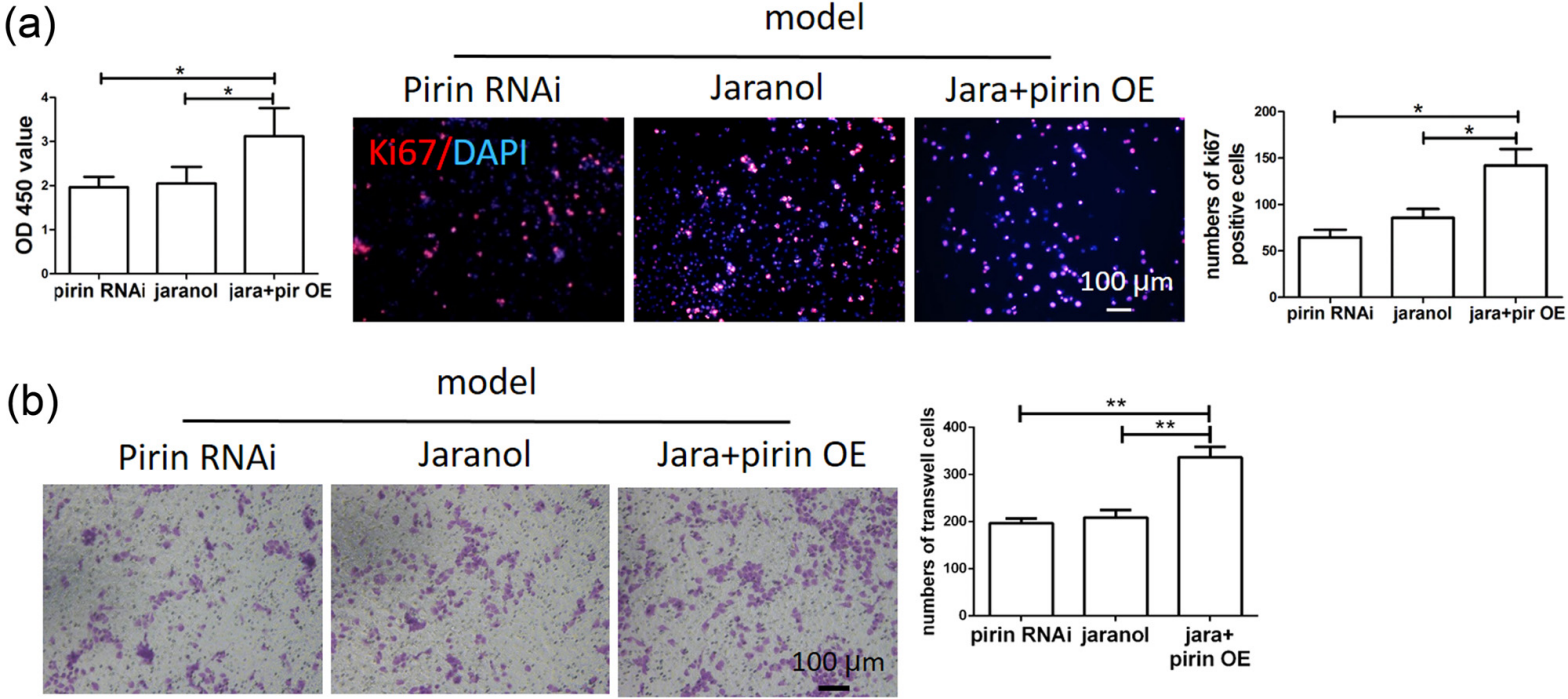
Jaranol inhibits mast cell proliferation and migration through Pirin. (a) CCK-8 and Ki67 immunofluorescence staining show that both Jaranol and interference with Pirin can inhibit mast cell proliferation, and the inhibitory effect of Jaranol on cell proliferation is weakened in mast cells overexpressing Pirin. (b) Transwell experiment shows that both Janroal and interference with Pirin can inhibit mast cell migration, and the inhibitory effect is weakened in mast cells overexpressing Pirin. Pir: Pirin; RNAi: interference; OE: overexpression. Scale bar: 100 μm. **p < 0.01.

We further investigated the effect of Jaranol and Pirin on mast cell function. As shown in Figure 6a, the results of the CCK-8 assay demonstrated that both interference with Pirin and the addition of Jaranol could inhibit mast cell proliferation. However, overexpression of Pirin reduced the inhibitory effect of Jaranol on mast cell proliferation. The Ki67 immunofluorescence assay also confirmed this observation, where the number of Ki67-positive cells remained low in both the Pirin interference and Jaranol groups. In contrast, in mast cells overexpressing Pirin and treated with Jaranol, the number of Ki67-positive cells significantly increased, indicating that Pirin promoted mast cell proliferation, and Jaranol partially inhibited Pirin-induced mast cell proliferation. Figure 6b shows that the migration ability of mast cells in the Pirin interference and Jaranol groups was lower, while overexpression of Pirin in mast cells treated with Jaranol significantly increased their migration ability. This suggests that Pirin promoted mast cell migration and that Jaranol partially inhibited Pirin-induced mast cell migration (*p* < 0.05).

### 3.7 Jaranol inhibited mast cell function by suppressing Pirin expression

**Figure 7:**
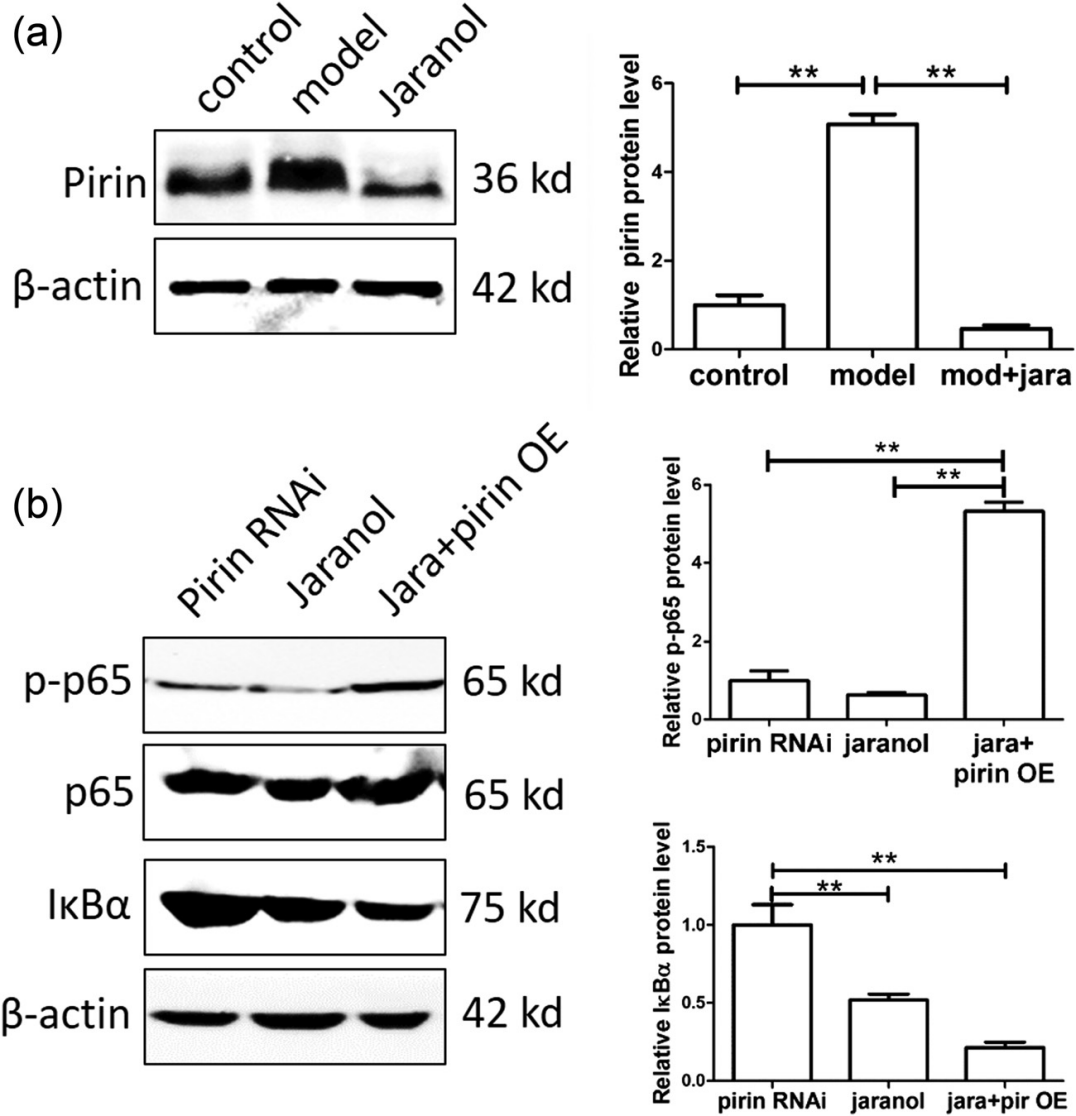
Jaranol inhibits mast cell function by suppressing Pirin expression. (a) WB shows that Jaranol inhibits Pirin expression. (b) WB results show that in mast cells overexpressing Pirin, adding Jaranol weakens the inhibitory effect on p65 phosphorylation and reduces IκBα expression, indicating that Jaranol promotes p65 phosphorylation through Pirin, thereby promoting mast cell-mediated inflammatory responses. RNAi: interference; OE: overexpression. **p < 0.01.

Based on the above results, Jaranol regulates the effect of Pirin on mast cell function. Therefore, we further investigated whether Jaranol affects mast cell function by regulating Pirin expression. The western blot (WB) results showed that Pirin expression was increased in the model group while adding Jaranol downregulated Pirin expression (Figure 7a). Both interference with Pirin and the addition of Jaranol inhibited p65 phosphorylation and promoted IκBα expression in mast cells. However, when mast cells overexpressing Pirin were treated with Jaranol, the inhibitory effect on p65 phosphorylation was weakened and IκBα expression was reduced, indicating that Jaranol’s inhibition of p65 phosphorylation was mediated by Pirin, thereby promoting mast cell-mediated inflammatory responses (Figure 7b).

## 4 Discussion

AR has been treated with Chinese herbal medicine for centuries and has been found to alleviate AR symptoms through immune modulation and anti-allergic or anti-inflammatory effects. Various clinical studies, including those on herbal treatments such as Yu-ping-feng San, Cure-allergic-rhinitis syrup, fermented red ginseng, and Biminne capsules, have been conducted to evaluate their efficacy [26]. In addition to oral administration, Chinese herbal medicine can be used to treat AR through nasal administration, and it can reduce the side effects of orally administered Chinese herbal medicine and improve safety.

We first established a mouse model of AR and performed HE staining on nasal respiratory tissues. The normal nasal respiratory epithelium includes a pseudostratified mucosal layer and submucosal tissue with blood vessels and glands. In the model group, HE staining showed that the mucosal tissue was significantly thickened with abundant inflammatory infiltration. After treatment with dexamethasone or Jaranol, the mice showed reduced nose-scratching symptoms, and HE staining showed a decrease in mucosal layer thickness and a significant decrease in inflammatory cell infiltration. Since mast cells are the main receptors in AR, mast cell staining showed that Jaranol reduced mast cell numbers in the nasal mucosa. Additionally, Ki67 staining of nasal mucosa suggested that Jaranol inhibited cell proliferation. These results suggest that Jaranol is effective in treating AR in mice. The early stage of AR is marked by the release of inflammatory factors by mast cells. Therefore, we conducted *in vitro* experiments using P815 mast cells. Real-time PCR and ELISA results showed that Jaranol inhibited the secretion of inflammatory factors (IFN-γ, TNF-α, IL-1β, IL-6, MCP-1, and CXCR10) in mast cells. Jaranol also inhibited mast cell proliferation and migration. In summary, this study confirmed *in vivo* and *in vitro* that Jaranol can treat AR. Research on Jaranol remains limited, and our study has enriched the potential indications of Jaranol.

Next, we explored the mechanism of Jaranol in treating AR. Using proteomic sequencing, we screened for differentially expressed and functional genes in the control, model, and Jaranol groups. Pirin, a nuclear protein with a significant role in inflammation and mast cell activation, showed notable differences in expression among the three groups. This suggests that Pirin could be a key target for Jaranol in treating AR. Further cell and animal experiments confirmed that Jaranol inhibits mast cell inflammatory factor secretion, proliferation, and migration, as well as mast cell function by targeting Pirin.

There is limited study on nasal administration of Chinese herbal medicine, and there is a lack of in-depth studies on individual herbs and compounds suitable for nasal delivery in traditional Chinese medicine formulations. The limitations of nasal delivery for Chinese herbal medicine include: (1) Many herbs used in nasal delivery have strong smells and can be irritating, which may cause nasal allergies or damage. (2) Nasal treatments often focus on symptom relief, leading to frequent recurrence of the illness. (3) Traditional nasal formulations are often made by grinding raw herbs or boiling them, resulting in low drug utilization. (4) In clinical studies, absorption enhancers in formulations can be toxic, strongly irritate the nasal mucosa, and affect ciliary function, leading to adverse reactions. (5) New formulations lack mature preparation methods, leading to unstable product quality and challenges in scaling up for industrial production, which limits the development of nasal delivery for Chinese medicine. The above reasons make it difficult to find suitable drugs for nasal administration. In our study, Jaranol is insoluble in water and can only be formulated as a nasal oil (which has a moisturizing effect), so side effects like nosebleeds and nasal dryness are not a major concern, additionally, Jaranol has a mild odor, is less irritating to the nasal cavity, and has good safety. However, the high viscosity of the oil formulation makes it less convenient for absorption and may affect mucosal absorption. Further optimization of the formulation process is needed to reduce these issues and improve patient use.

Moreover, this study did not explore the safety of Jaranol. An acute toxicity study showed that a single oral dose of Jaranol did not cause any lethal or general behavioral changes in mice. In terms of subacute toxicity, oral administration of 200 mg/kg BW/day of Jaranol significantly affected red blood cells and liver and kidney function in rats [27]. Liver and kidney function damage is a common side effect of Chinese herbal medicine, so to avoid this, our study recommends using Jaranol as nasal drops. Common side effects of clinical nasal drops are nosebleeds, nasal dryness, and long-term use leading to drug resistance. Our study did not find nosebleeds and nasal dryness in the AR mouse model. However, further investigation is needed to determine whether long-term use of Jaranol leads to drug resistance.

## 5 Conclusion

Jaranol can reduce inflammation and histopathological changes associated with AR, by inhibiting Pirin expression in mast cells, Jaranol presents a promising natural nasal drop for treating AR. However, Jaranol nasal drops are currently in an oil formulation.

In future studies, we will further improve the preparation process to enhance the comfort of clinical use.
